# Adaptive Optics Tip-Tilt Correction Based on Smith Predictor and Filter-Optimized Linear Active Disturbance Rejection Control Method

**DOI:** 10.3390/s23156724

**Published:** 2023-07-27

**Authors:** Lingxi Kong, Kangjian Yang, Chunxuan Su, Sicheng Guo, Shuai Wang, Tao Cheng, Ping Yang

**Affiliations:** 1 Key Laboratory on Adaptive Optics, Institute of Optics and Electronics, Chinese Academy of Sciences, Chengdu 610209, China; 2 Institute of Optics and Electronics, Chinese Academy of Sciences, Chengdu 610209, China; 3 School of Electronic, Electrical and Communication Engineering, University of Chinese Academy of Sciences, Beijing 100049, China

**Keywords:** adaptive optics, tip-tilt mirror, Smith predictor, linear active disturbance rejection

## Abstract

A tip-tilt mirror (TTM) control method is designed to enhance the control bandwidth and ensure the rejection performance of the adaptive optics (AO) tip-tilt correction system. Optimized with the Smith predictor and filter, linear active disturbance rejection (LADRC) is adopted to achieve the tip-tilt correction. An AO tip-tilt correction experimental platform was built to validate the method. Experimental results show that the proposed method improves the control bandwidth of the system by at least 3.6 times compared with proportional–integral (PI) control. In addition, under the same control bandwidth condition, compared with the Smith predictor and proportional–integral (PI–Smith) control method, the system is more capable of rejecting internal and external disturbances, and its dynamic response performance is improved by more than 29%.

## 1. Introduction

AO is the technology for correcting dynamic optical wavefront aberration by detecting and compensating wavefront aberrations in real time [1]. In the field of laser-beam cleaning, AO has the advantage of improving the far-field beam quality of output lasers [2]. In the process of laser-beam cleaning, correcting the tip-tilt aberration is the primary task to stabilize the beam pointing in the detector field of view during the AO closed-loop process [3,4,5]. However, a high-frequency tip-tilt disturbance is introduced because of thermal effects, material defects, and the diversification of AO application scenarios [6]. It affects the laser output beam pointing, making it difficult to stabilize the beam pointing in a stable region. The tip-tilt disturbance leads to severe degradation of the far-field beam quality of the laser, which limits the application of the laser [7].

There are two challenges in AO tip-tilt correction. On the one hand, the time delay caused by the existence of data acquisition and control calculations limits the system control bandwidth [8,9]. On the other hand, the dynamic response performance of a TTM is sensitive to the effects of internal and external disturbances [10,11,12]. The change in the time-delay parameter of the controlled model mainly causes internal disturbances. External disturbances include the vibration of the experimental devices and the instability of the application scenario of the AO system. These two challenges introduce high-frequency tilt disturbances to the system, which exceed the dynamic response capability of the tip-tilt mirror and cause the beam to point away from the detector’s field of view [13,14]. Therefore, the impact of the time delay and disturbances on the tip-tilt correction performance of the system needs to be compensated simultaneously to achieve high bandwidth and high dynamic response TTM control in the AO system.

Recently, some control methods were proposed to compensate for the effect of the time delay on the tip-tilt correction performance of AO. Luo et al. [15] embedded the wind speed estimation and prediction control into the AO system and established the control procedure for wind speed estimation and prediction algorithm in the closed loop. They used a combination of geometric interpolation and prediction for compensation correction of the restoration voltage, which reduced the root mean square of the corrected residuals by about 10% and decreased the correction error due to the time delay. However, the method is computationally intensive and requires high computational power on real-time processors. Ruan et al. [16] improved the closed-loop correction performance of the system using a multiple closed-loop control method with limited bandwidth, considering the time-delay limitation. However, the technique failed to improve the system control bandwidth in essence. Based on classical PI control, Li et al. [8] performed Smith prediction to compensate for the effect of time delay on the system correction performance. Smith enhanced the system control bandwidth, but PI control experienced challenges in overcoming the impact on the system correction performance because of the mismatch between the Smith compensation parameter and the system time-delay parameter, which was constrained by internal disturbances. In addition, control methods for disturbance rejection were widely researched. Zhao et al. [17] used a robust control method to design a controller for the AO system. They minimized the H_2_ norm under the H_ꝏ_ norm constraint, effectively suppressing the low-frequency disturbances. However, the method was computationally extensive, which again exacerbated the degree of time delay of the AO system in another way. LQG control [18] and hybrid control [19,20] can effectively identify and reject high-frequency tip-tilt disturbances. Still, the control performance depended on the accuracy of the model establishment, and the system identified the jitter of the beam pointing in real time, which required hard real time and the accuracy of the calculation. Li et al. [21] improved the disturbance rejection and tracking performance of the TTM in optical communication systems by optimizing the output estimation of the system with an improved LADRC method. Still, the effect of internal disturbances was not considered in their work. The control methods mentioned above improve the tip-tilt correction effect but fail to simultaneously address the influence of the time delay and disturbances on the system calibration performance.

To provide a simultaneous solution for the effect of the time delay and disturbances on the correction performance of the system, in our previous work, we innovatively proposed a TTM control method with a Smith predictor and filter-optimized linear active disturbance rejection (FLADRC–Smith) [22]. We used a Smith predictor to enhance the control bandwidth of the system and designed a filtering unit to suppress internal and external disturbances. Simulation results showed that the method improved the system’s control bandwidth and dynamic response performance. 

This paper analyzes the influence of FLADRC–Smith control parameter selection on the system bandwidth and noise rejection performance according to the equivalent mathematical model of the AO tip-tilt correction system. Finally, the effectiveness of the TTM control method with FLADRC–Smith is verified by building an AO tip-tilt correction experimental platform.

## 2. AO Tip-Tilt Correction Principle Based on the FLADRC–Smith Control

### 2.1. Mathematical Model of AO Tip-Tilt Correction

**Figure 1:**
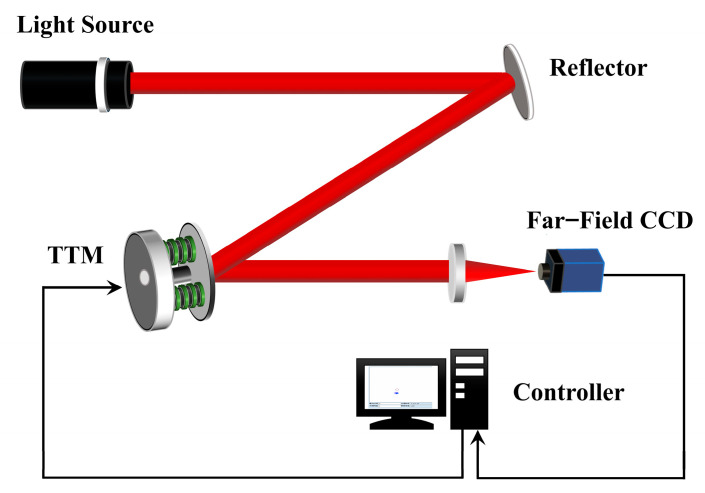
Schematic diagram of the AO tip-tilt correction system.

The schematic diagram of the AO tip-tilt correction system is shown in Figure 1. A far-field CCD detects tip-tilt disturbances and feeds back to the controller, which calculates the control voltage and then applies it to the TTM to drive a slight angle deflection, thus compensating the tip-tilt disturbance and stabilizing the far-field spot in the CCD field of view. As an actuator for correction, the TTM produces mechanical resonance phenomena in the high-frequency band because of its inherent elastic structure. However, the first resonance frequency is in the hundreds and thousands of hertz. Therefore, in the frequency range of concern for the system, its ideal transfer function is as follows:

(1) $$\mathrm{TTM}\left(s\right)=1,$$

Combined with the τ-second time delay present in the system, the equivalent transfer function of the system can be expressed as follows:

(2) $$P_{0}\left(s\right)=\mathrm{TTM}\left(s\right)*e^{-\tau s}=e^{-\tau s},$$

### 2.2. Design of FLADRC–Smith Control Method

**Figure 2:**
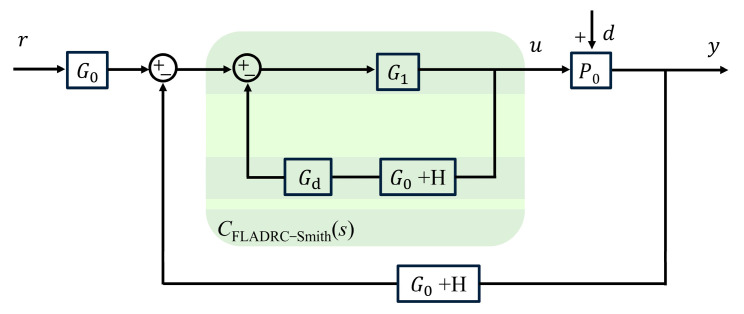
Block diagram of AO tip-tilt correction control using FLADRC–Smith.

Figure 2 shows the control block diagram of an AO tip-tilt correction system using the FLADRC–Smith control. In Figure 1, *r* is the set value of the TTM deflection angle, *d* is the external disturbance signal, *u* is the voltage control signal of TTM, *y* is the output deflection angle of TTM, and *y* has a time delay of τ seconds in the time domain concerning *u*. The feedback signal comes from the TTM deflection angle measurement sensor, and the error of the deflection angle is sent to the controller.

In Figure 2:

(3) $$\left\{\begin{array}{l} G_{0}=k_{p}+\frac{k_{i}}{s}\\ G_{1}=\frac{\omega_{c}{\left(s+\omega_{0}\right)}^{3}}{s^{4}+\left(3\omega_{0}+\omega_{c}\right)s^{3}+\left(3\omega_{0}^{2}+3\omega_{0}\omega_{c}\right)s^{2}+(\omega_{0}^{3}+3\omega_{0}^{2}\omega_{c})s}\\ H=\frac{\omega_{0}^{3}}{b_{0}}*\frac{s^{2}}{{\left(s+\omega_{0}\right)}^{3}} \end{array},\right.$$

where $\omega_{0}$ is the bandwidth of the extended state observer in rad/s in LADRC, $k_{p}$ and $k_{i}$ are the feedback control law parameters, and $\omega_{c}$ is the cutoff frequency of the filtering unit. $G_{d}\left(s\right)$ is the Smith predictor model designed according to Equation (2):

(4) $$G_{d}\left(s\right)=1-e^{-\tau_{1}s},$$

where $\tau_{1}$ is the compensation parameter of the Smith predictor. $G_{0}$ and H combine to form the deflection angle measurement sensor, and the controller consists of $G_{0}$, the deflection angle measurement sensor, and $G_{d}\left(s\right)$. Accordingly, the closed-loop transfer function $M_{\mathrm{FLADRC}-\mathrm{Smith}}\left(s\right)$ of the control loop can be obtained as:

(5) $$M_{\mathrm{FLADRC}-\mathrm{Smith}}\left(s\right)=\frac{G_{0}G_{1}P_{0}}{1+G_{1}^{\prime }\left(G_{0}+H\right)\left(P_{0}+G_{d}\right)}=\frac{\omega_{c}{\left(s+\omega_{0}\right)}^{3}\left(k_{p}s+k_{i}\right)e^{-\tau s}}{B\left(s\right)},$$

where

(6) $$\begin{array}{l} B\left(s\right)=s^{2}\left[s^{3}+\left(3\omega_{0}+\omega_{c}\right)s^{2}+\left(3\omega_{0}^{2}+3\omega_{0}\omega_{c}\right)s+\left(\omega_{0}^{3}+3\omega_{0}^{2}\omega_{c}\right)\right]+\\ \left[\omega_{c}{\left(s+\omega_{0}\right)}^{3}\left(k_{p}s+k_{i}\right)+\frac{\omega_{0}^{3}\omega_{c}}{b_{0}}s^{3}\right]\left[e^{-\tau s}+\left(1-e^{-\tau_{1}s}\right)\right] \end{array},$$

The characteristic equations of the closed-loop transfer function are processed using the pole placement method. When set up, $b_{0}\gg \omega_{0}^{3}$ and $k_{i}\to 0$, $B\left(s\right)$ can be factorized into $B\left(s\right)=\left(s+k_{i}/k_{p}\right)C\left(s\right)$ using the synthetic division method. To ensure that $B\left(s\right)$ has strong disturbance rejection in the high-frequency band, and then by finding the rational roots of polynomials using the irreducible fraction, $C\left(s\right)$ can be factorized into $C\left(s\right)=\left(s+\frac{3}{2}\omega_{0}\right)D\left(s\right)$. When $D\left(s\right)$ is expanded to $D\left(s\right)=\left(s+k_{p}\omega_{c}\right)E\left(s\right)$, a scaling factor 0<μ<1 is introduced to obtain the desired pole as $\mu \omega_{0}$, where $\omega_{c}\approx \mu \frac{\omega_{0}}{k_{p}}$. When designing the FLADRC–Smith controller in the AO tilt correction system, the power spectral density of the disturbance signal should be analyzed to determine the distribution band of the disturbance signal to confirm the desired closed-loop pole $\mu \omega_{0}$ and then adjust $k_{p}$ according to the actual response performance of the system.

### 2.3. Bandwidth Analysis

The error transfer function reflects the steady-state error of the control system and characterizes the system’s ability to suppress the error signal. The larger the error attenuation bandwidth, the stronger the rejection capability of the system. Therefore, to analyze the disturbance rejection performance of the control system, it is necessary to analyze the error attenuation bandwidth of the system.

**Figure 3:**
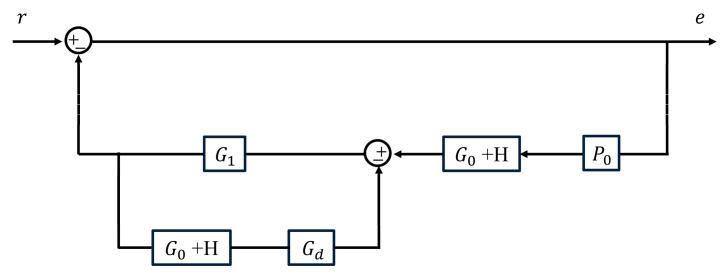
Block diagram of the connection between the input signal and the error signal.

The block diagram of the relationship between the system inputs and the error is shown in Figure 3, which means the error transfer function:

(7) $$M_{\mathrm{ef}}\left(s\right)=\frac{1+G_{1}\left(G_{0}+H\right)G_{d}}{1+G_{1}\left(G_{0}+H\right)\left(P_{0}+G_{d}\right)}=\frac{1+G_{1}\left(G_{0}+H\right)G_{d}}{1+G_{1}\left(G_{0}+H\right)}=\frac{B^{\prime }\left(s\right)}{B\left(s\right)},$$

where

(8) $$B^{\prime }\left(s\right)=s^{2}\left(s+\frac{7}{5}\omega_{0}\right)\left[s^{2}+\left(\frac{80}{49}\omega_{0}+\frac{30}{49}\omega_{c}\right)s+\left(\frac{5}{7}\omega_{0}^{2}+\frac{15}{7}\omega_{0}\omega_{c}\right)\right],$$

Comparing Equation (7) with Equation (5), it can be observed that the closed-loop transfer function of the AO tip-tilt correction control system has the same pole composition as the error transfer function. When

(9) $$\omega_{c}\approx \mu \frac{\omega_{0}}{k_{p}},$$

is set up, the error attenuation bandwidth of the control system is $\mu \omega_{0}$ because of the approximate elimination of the zero value $-\frac{7}{5}\omega_{0}$ and the polar component $-\frac{3}{2}\omega_{0}$. The rejection performance of the system can be evaluated according to the error attenuation bandwidth.

### 2.4. Noise Analysis

**Figure 4:**
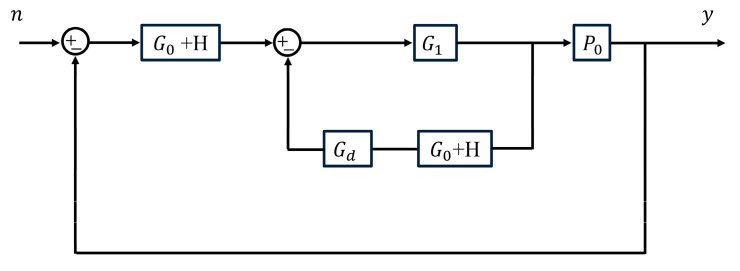
The role of noise in the system output signal.

There are many contradictions in process control, such as the conflict between speed and stability. Similarly, there is a contradiction between control bandwidth and system noise rejection capability. The larger the control bandwidth, the more measurement noise is inevitably introduced while correcting for external disturbances. In Figure 4, the noise *n* is shown on the system output of the schematic diagram. Accordingly, the noise transfer function can be derived as:

(10) $$M_{\mathrm{nf}}\left(s\right)=\frac{G_{1}\left(G_{0}+H\right)P_{0}}{1+G_{1}\left(G_{0}+H\right)\left(P_{0}+G_{d}\right)}=\frac{G_{1}\left(G_{0}+H\right)P_{0}}{1+G_{1}\left(G_{0}+H\right)}=\frac{A\left(s\right)}{B_{1}^{\prime }\left(s\right)},$$

where

(11) $$A\left(s\right)=\left[\omega_{c}{\left(s+\omega_{0}\right)}^{3}\left(k_{p}s+k_{i}\right)+\frac{\omega_{0}^{3}\omega_{c}}{b_{0}}s^{3}\right]e^{-\tau s},$$

(12) $$\begin{array}{l} B_{1}^{\prime }\left(s\right)=s^{2}\left[s^{3}+\left(3\omega_{0}+\omega_{c}\right)s^{2}+\left(3\omega_{0}^{2}+3\omega_{0}\omega_{c}\right)s+\left(\omega_{0}^{3}+3\omega_{0}^{2}\omega_{c}\right)\right]\\ +\left[\omega_{c}{\left(s+\omega_{0}\right)}^{3}\left(k_{p}s+k_{i}\right)+\frac{\omega_{0}^{3}\omega_{c}}{b_{0}}s^{3}\right] \end{array},$$

Considering only the amplitude–frequency characteristics of the noise transfer function, it is noted that $M_{\mathrm{nf}}\left(s\right)$ contains four zeros and five poles. According to the effect of pole–zero on the frequency domain characteristics of the system, it is known that the amplitude–frequency characteristic curve of $M_{\mathrm{nf}}\left(s\right)$ eventually decreases at a rate of −20 dB/dec. Therefore, the system has the ability to suppress noise. Define the frequency corresponding to the amplitude–frequency curve over the −3 dB gain point as the noise attenuation bandwidth. It can be seen that the smaller the value, the stronger the system’s ability to suppress noise.

The above analysis shows that the system’s control bandwidth and noise suppression capability conflict with each other. To ensure the noise rejection capability of the system, the bandwidth of the system should not be too large. When selecting the control parameters for the FLADRC–Smith controller, the disturbance rejection performance of the system and the control index requirements should be considered comprehensively.

## 3. Results

### 3.1. AO Tip-Tilt Correction Experimental Platform

**Figure 5:**
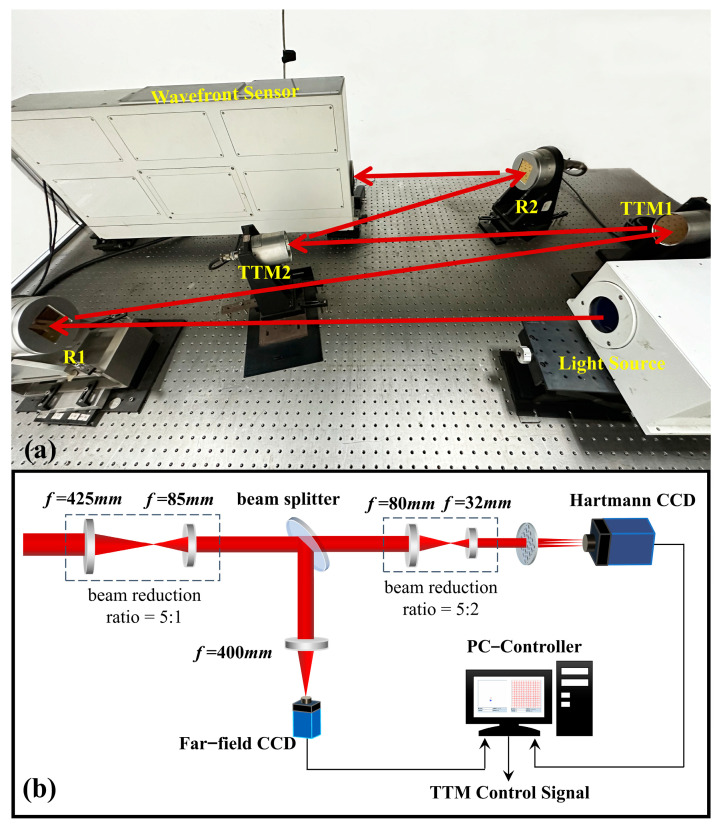
Experimental platform for AO tip-tilt correction: (**a**) Experimental platform; (**b**) Schematic diagram of Hartmann.

The AO tip-tilt correction experimental platform is shown in Figure 5. 

In Figure 5a, the light source wavelength is 635 nm. Before entering the wavefront sensor, the output beam passes through reflector 1 (R1), disturbance TTM1, correction TTM2, and R2. In particular, TTM1 and TTM2 have an aperture of 70 mm, a resonance frequency of 1000 Hz, and a dynamic response range of ±100 μrad. Figure 5b shows the schematic diagram of the wavefront sensor, which contains the Hartmann wavefront sensor, the far-field CCD, and the beam splitter with a sampling frequency of 200 Hz. In this experimental platform, the signal generator controls the Ethernet interface high-voltage amplifier to generate a control voltage with disturbances, which drives TTM1 to produce a slight amplitude deflection and makes the beam point jitter in the y-direction. TTM2 and the deformable mirror are driven by a fiber-optic high-voltage amplifier sending control voltage with a voltage limit of ±2 V. The control algorithm enables TTM2 to reject the disturbance generated by TTM1, and the deformable mirror corrects the residual wavefront aberration.

**Figure 6:**
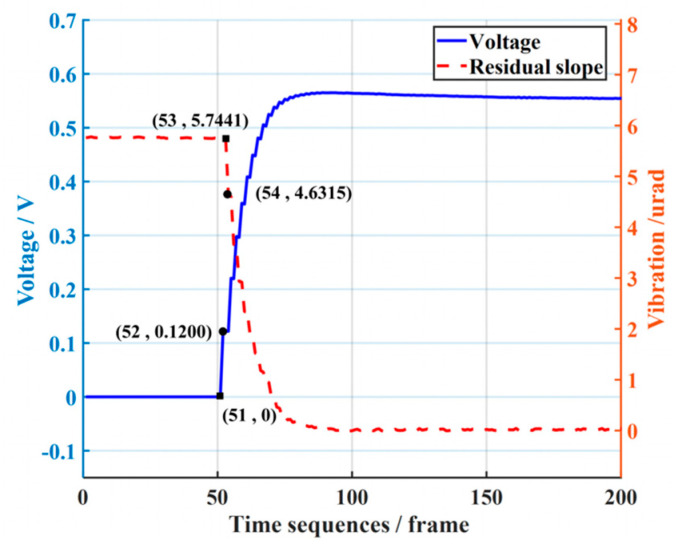
Analysis of time delay.

To measure the time delay of the experimental platform, the static tip-tilt aberration is corrected. As seen from the correction results in Figure 6, the control voltage is applied to TTM2 in the 52nd of the time sequence, but in the 54th, the tip-tilt aberration starts to be compensated. This indicates that there is a time delay τ in the control system, which is two times the sampling period. Accordingly, the time-delay compensation parameter $\tau_{1}$ in the Smith predictor is set to 0.01 s during the experiment.

### 3.2. Comparison with PI Control Method

To verify the advantages of the FLADRC–Smith method in enhancing the control bandwidth when applied to the AO tip-tilt correction system, the FLADRC–Smith control method is compared with the PI control method on the experimental platform [23].

**Figure 7:**
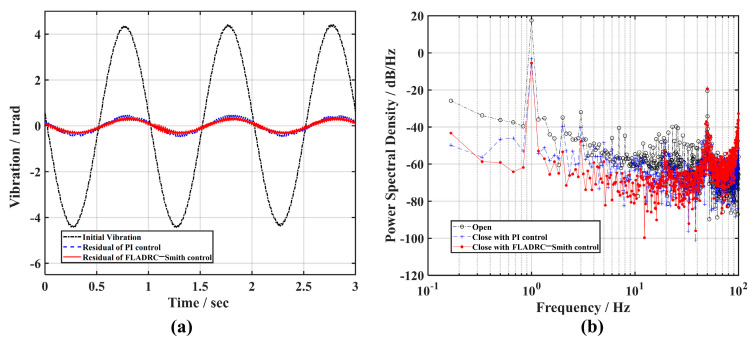
The comparison of the suppression results of 1 Hz vibration: (**a**) time domain; (**b**) frequency domain.

First, a sine wave signal with an amplitude of 1 V and a frequency of 1 Hz is generated using a signal generator and applied to the *y*-direction of TTM1 through an Ethernet interface high-voltage amplifier. At this time, the output beam of the light source produces a sine wave disturbance in the *y*-direction, as shown by the dotted line in Figure 7a. The power of this disturbance at 1 Hz is 17.5 dB/Hz, as can be seen from the dotted line marked by the circle in Figure 7b.

Combined with manual experience and considering the bandwidth of the control system, the dynamic response performance of the TTM, and the need to avoid unstable phenomena such as overshooting in the closed-loop process, the control parameters of the PI controller are set to $K_{p}=0$, $K_{i}=40$. The residual tip-tilt aberration of TTM2 after correction is shown as dashed lines in Figure 7a, corresponding to the power spectral density curves illustrated with plus dotted lines in Figure 7b. The disturbance power of the tip-tilt aberration signal at 1 Hz is below 0 dB, which tells us that the sine disturbance is effectively corrected. The control parameters of the FLADRC–Smith controller are tuned to guarantee that TTM2 has the same rejection capability for disturbances as when PI control is used, as shown by the solid line in Figure 7a and the solid line marked by dots in Figure 7b, where the control parameters are set to $\omega_{0}=250$ rad/s, μ=0.6, $k_{p}=16$. With the parameter settings, the ideal error-attenuation bandwidth of the system is, theoretically, 6.25 Hz maximum for PI control and 23.87 Hz maximum for FLADRC–Smith control.

**Figure 8:**
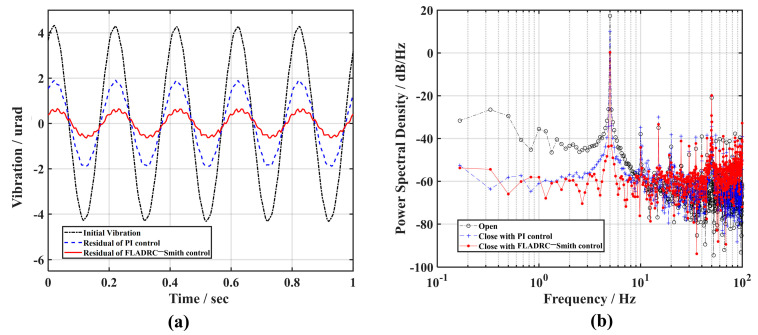
The comparison of the suppression results of 5 Hz vibration: (**a**) time domain; (**b**) frequency domain.

Next, holding the control parameters of the controller constant, the frequency of the signal generator is increased to 5 Hz, and Figure 8 shows the correction results. In Figure 8a, it can be seen that the amplitude of the tip-tilt aberration becomes larger under PI control compared to the suppression effect on the 1 Hz frequency disturbance signal, while the control of TTM2 using the FLADRC–Smith method still better suppresses the disturbance. As can be seen in Figure 8b, the power of the disturbance signal decreases from 17.5 dB/Hz to below 0 dB at 5 Hz frequency under FLADRC–Smith control. At the same time, it is greater than 10 dB/Hz under PI control, which provides poor rejection of the 5 Hz disturbance signal, indicating that the control bandwidth of the system is below 5 Hz.

**Figure 9:**
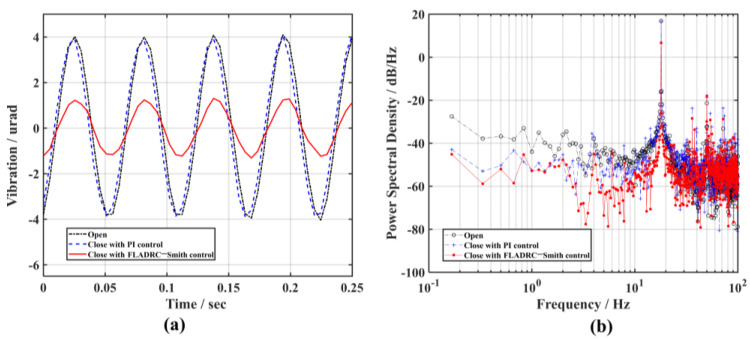
The comparison of the suppression results of 18 Hz vibration: (**a**) time domain; (**b**) frequency domain.

**Figure 10:**
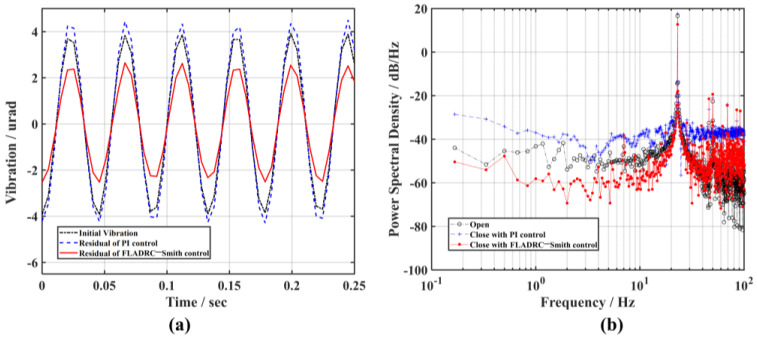
The comparison of the suppression results of 23 Hz vibration: (**a**) time domain; (**b**) frequency domain.

Finally, to explore the control bandwidth of the system during FLADRC–Smith control, the controller parameters are kept constant, the sine disturbance frequency of the signal generator output is increased, and its ability to suppress high-frequency disturbance signals is analyzed. Figure 9 and Figure 10 show the rejection results for 18 Hz and 23 Hz sine disturbance signals under FLADRC–Smith and PI control, respectively. The comparison shows that TTM2 has difficulty suppressing the 18 Hz sine disturbance under PI control and even indicates the counter effect of amplifying the disturbance for the 23 Hz sine signal. According to Figure 9a, the suppression of the disturbance amplitude by FLADRC–Smith is evident in the time domain, and the power of the disturbance component is suppressed to 6.7 dB/Hz, as shown in Figure 9b. However, the results in Figure 10 show that the FLADRC–Smith control becomes less effective in suppressing the 23 Hz sine disturbance, indicating that the control bandwidth of the system is between 18 Hz and 23 Hz, which is consistent with the results of the theoretical calculations.

By analyzing the suppression effect of different frequency sine disturbance signals above, we can observe that the control bandwidth of the system is between 18 Hz and 23 Hz when the FLADRC–Smith control method is used. In contrast, driving TTM2 to suppress the disturbance signal at 5 Hz under PI control is challenging, and the control bandwidth is less than 5 Hz.

In summary, the FLADRC–Smith control method can reject high-frequency disturbance signals better than the PI control method. It improves the control bandwidth of the system by at least 3.6 times.

### 3.3. Comparison with PI–Smith Control Method

The Smith predictor is sensitive to the change in the time-delay parameter during the system correction process. Therefore, to further verify the system’s disturbance rejection performance and dynamic response performance when the FLADRC–Smith control method is used, the FLADRC–Smith control method is compared with the PI–Smith control method in the experiment.

**Figure 11:**
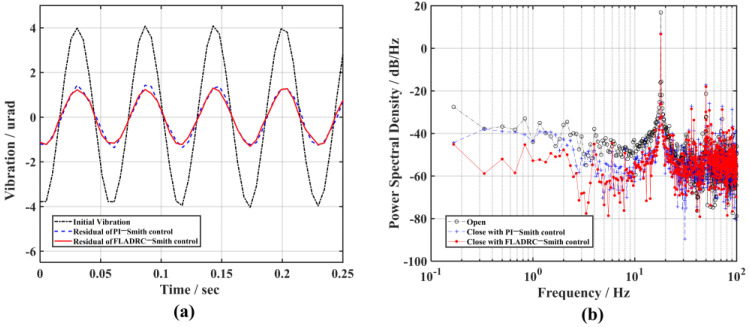
Validation of bandwidth enhancement capability at 18 Hz: (**a**) time domain; (**b**) frequency domain.

**Figure 12:**
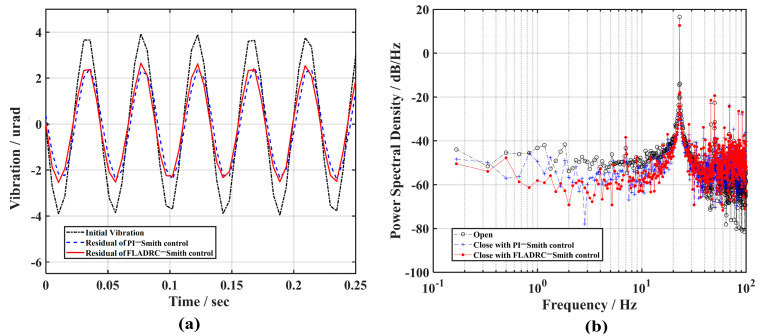
Validation of bandwidth enhancement capability at 23 Hz: (**a**) time domain; (**b**) frequency domain.

During the experiments, the control parameters of the FLADRC–Smith controller are followed, that is, $\omega_{0}=250$ rad/s, μ=0.6, and $k_{p}=16$. Then, adjust the control parameters of PI–Smith to $K_{p}=0$, $K_{i}=84$ so that the system control bandwidth is the same under both control methods. With this parameter setting, the suppression effects of the two control methods for high-frequency disturbances at 18 Hz and 23 Hz are shown in Figure 11 and Figure 12. Figure 11 and Figure 12 illustrate that the system has the same control bandwidth and rejection capability for high-frequency disturbances under FLADRC–Smith and PI–Smith control.

**Figure 13:**
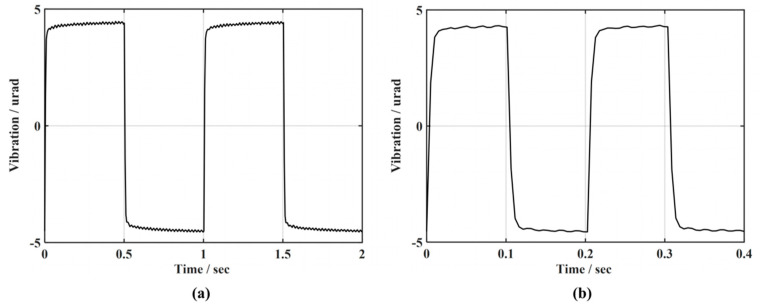
Square-wave disturbance signal: (**a**) 1 Hz; (**b**) 5 Hz.

To more visually compare the dynamic response capabilities of FLADRC–Smith and PI–Smith when suppressing disturbances, a signal generator is used to generate square-wave signals with frequencies of 1 Hz and 5 Hz, respectively, which are applied to TTM1 through an Ethernet interface high-voltage amplifier, where the duty cycle of the square-wave signal is 50%, as shown in Figure 13. In the experiment, the setting time required to recover ±5% of the steady-state value of the response of TTM2 is used as the dynamic response performance evaluation index.

**Figure 14:**
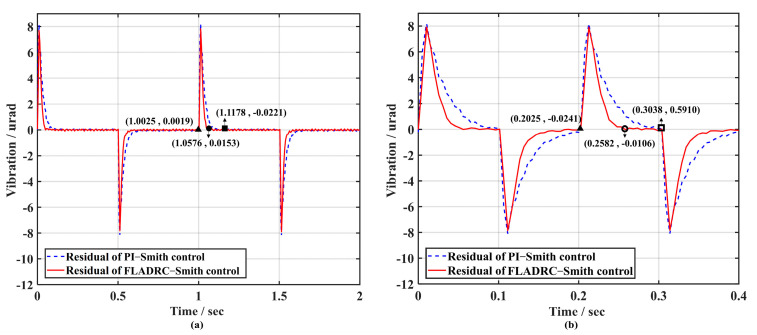
Comparison of dynamic response performance of disturbance rejection: (**a**) 1 Hz; (**b**) 5 Hz.

Figure 14 compares the results of TTM2 for suppressing 1 Hz and 5 Hz square-wave disturbance signals driven by FLADRC–Smith and PI–Smith. It can be seen that the system produces a transient response with both control methods at the rising and falling edges of the square-wave signal, and the tip-tilt disturbance can be quickly suppressed. When suppressing the 1 Hz square-wave signal, the setting time for PI–Smith control of TTM2 to resume stable tracking is 0.1153 s. In contrast, the setting time for FLADRC–Smith control is 0.0551 s, which improves the dynamic response performance by 52.2% compared to PI–Smith, as shown in Figure 14a. The setting time for FLADRC–Smith control when suppressing a 5 Hz square-wave signal is 0.0557 s, which improves the dynamic response performance by 45.1% compared to PI–Smith, as shown in Figure 14b.

**Figure 15:**
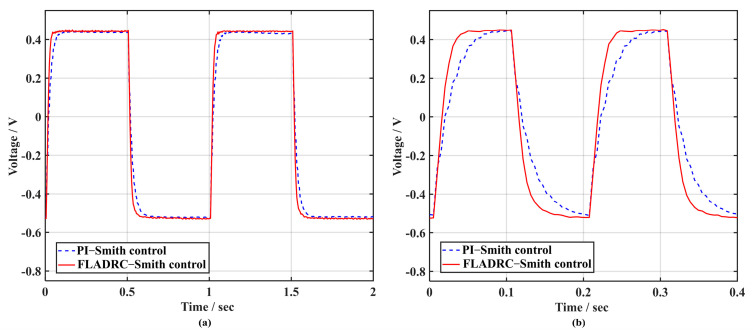
Comparison of dynamic response performance of voltage: (**a**) 1 Hz; (**b**) 5 Hz.

Further analysis of the voltages of the two control methods when driving TTM2 to suppress 1 Hz and 5 Hz square-wave signals, as shown in Figure 15, indicates that the voltage response of TTM2 is faster using FLADRC–Smith when the rising and falling edges of the square-wave movement come in.

**Figure 16:**
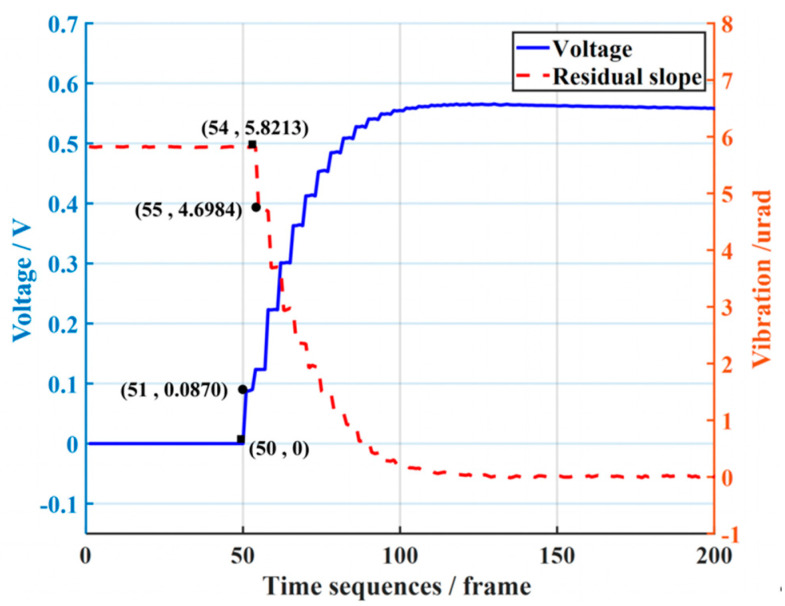
Correction of the time-delay system with 4 times sampling period.

**Figure 17:**
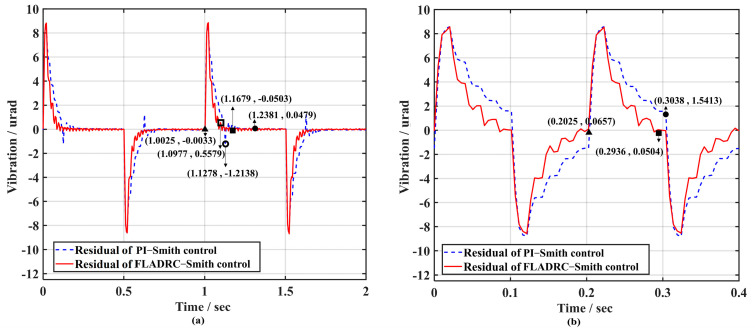
Dynamic response performance comparison at 4 times sampling period delay: (**a**) 1 Hz; (**b**) 5 Hz.

Theoretically, according to the setting of Smith predictor parameters, the maximum time-domain boundary of the internal disturbance that the Smith predictor can stably suppress is four times the sampling period. The control parameters are kept constant to compare the two controllers’ robustness. That is, $\tau_{1}$ is 0.01 s for both control methods. Increase τ to four times the sampling period, that is, 0.02 s, at which time the closed-loop state of the AO tip-tilt correction experimental platform is shown in Figure 16. With a delay time of four times the sampling period, PI–Smith and FLADRC–Smith are used to drive TTM2 to suppress the square-wave disturbance signal, as shown in Figure 13, and Figure 17 shows the correction results.

**Figure 18:**
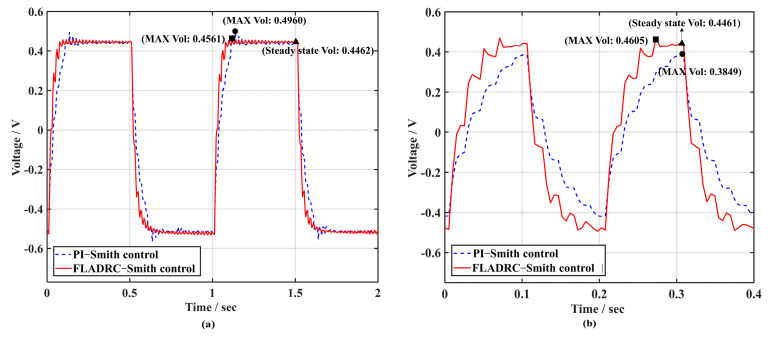
Voltage comparison at 4 times sampling period delay: (**a**) 1 Hz; (**b**) 5 Hz.

According to Figure 17a, the rising and falling edge moments of the 1 Hz square-wave disturbance signal’s peak error generated by TTM2 during PI–Smith control is 1.2138 μrad (the hollow circle mark). The setting time to recover the stable correction is 0.2356 s (the solid circle mark). At the same time, FLADRC–Smith drives TTM2, producing a peak error of 0.5579 μrad (the hollow square marker). It can recover the stable correction in 0.1654 s (the solid square marker), improving the dynamic response performance of the system by 29.8%. Once the square-wave signal frequency is raised to 5 Hz, PI–Smith cannot suppress the tip-tilt disturbance to 0 μrad at the duty cycle set by the square wave when the rising and falling edges come. Still, the system can be restored to a stable tracking control state within 0.0911 s using FLADRC–Smith, which significantly improves the dynamic response performance of the system. The voltage applied to TTM2 under the two control methods is then analyzed as shown in Figure 18.

As can be observed in the dashed line in Figure 18a, the PI–Smith control has a large overshoot at the rising and falling edges, with a peak overshoot of 11.16%. The FLADRC–Smith control method shown in the solid line has a peak overshoot of only 2.22%, reducing the shock to TTM2 due to voltage overshoot during the tip-tilt correction process. Additionally, as can be seen in Figure 18b, the control voltage calculated with PI–Smith is small compared to that calculated with FLADRC–Smith for TTM2, making correcting the 5 Hz square-wave disturbances difficult.

Figure 14 and Figure 17 indicate that in the absence of internal perturbations, TTM2 generates less overshoot, suppresses more quickly, and restores the stable correction state when TTM2 is controlled using FLADRC–Smith compared to PI–Smith, improving the dynamic response performance of the system. When the internal disturbance reaches the maximum boundary in the theoretical time domain, the PI–Smith becomes less able to suppress the disturbance because of the difficulty of the PI in compensating for the need of the Smith predictor for the accuracy of the time-delay parameter. At the same time, the FLADRC–Smith can guarantee the complete suppression of the disturbance. 

In addition, according to the comparison of the control voltages of TTM2 calculated using the two control methods in Figure 15 and Figure 18, when FLADRC–Smith is used, the overshoot of the transition process of the control voltage is slight in the moments of the rising and falling edges of the square-wave signal, which reduces the transient shock to TTM2 due to the sudden change in voltage during the tip-tilt correction process.

## 4. Conclusions

This paper adopts the FLADRC–Smith control method for the AO tip-tilt correction experimental platform using the mathematical model of the AO system. A detailed performance analysis was performed, and comparisons were conducted with the PI and PI–Smith control methods. The experimental results show that the FLADRC–Smith method enhances the control bandwidth of the system by at least 3.6 times compared with PI control, which improves the dynamic response performance of the control system compared with the PI–Smith control method. The FLADRC–Smith method achieves tip-tilt correction control with high bandwidth and high dynamic response performance, which is advantageous for applications in laser-beam cleaning, astronomical observation, etc.

## Data Availability

The data presented in this study are available on request from the corresponding author. The data are not publicly available for privacy reasons.

## Abbreviations

The following abbreviations are used in this manuscript:

AO	adaptive optics
TTM	tip-tilt mirror
PI	proportional–integral
PI–Smith	Smith predictor and proportional–integral
LADRC	linear active disturbance rejection
FLADRC–Smith	Smith predictor and filter-optimized linear active disturbance rejection
